# Effect of Different Dominant Phase on NO_2_-Sensing Properties of ZnO/SnO_2_ Composites

**DOI:** 10.3390/mi17101115

**Published:** 2026-09-24

**Authors:** Jipeng Zhao, Zhigang Tao, Zhenyue Tu, Hongxia Bian, Jiankang Huang

**Affiliations:** 1 Gansu Province Special Equipment Inspection and Testing Institute, Lanzhou 730050, China; 2 College of Science, Gansu Agricultural University, Lanzhou 730070, China; 3 School of Materials Science and Engineering, Lanzhou University of Technology, Lanzhou 730050, China

**Keywords:** NO_2_ sensing, gas sensor, dominant phase, ZnO, SnO_2_

## Abstract

The influence of different dominant phases on the gas-sensing properties of metal oxide n-n heterojunctions remains unclear. In this work, two kinds of phase-inverted ZnO/SnO_2_ heterojunction composites (SZ and ZS series) are fabricated via a facile solid-state grinding method. Characterization results show that ZS-7 exhibits finer and more uniform grains with a specific surface area of 70.30 m^2^·g^−1^. SZ-7 has a high oxygen vacancy content of 26.28%, and its Sn 3d characteristic peaks shift entirely toward higher binding energies, which verifies electron redistribution at the n-n heterojunction interface. Under UV irradiation, room-temperature NO_2_ gas-sensing tests reveal that the ZS series exhibits overall superior performance to the SZ series. ZS-7 delivers a gas response of 46.49, with response/recovery times of 35 s/122 s and an ultralow detection limit of 74 ppb. Mechanism analysis indicates that the ZnO/SnO_2_ heterojunction facilitates the separation and migration of photogenerated carriers under UV irradiation. The phase-inverted structure further accelerates photogenerated carrier separation and optimizes charge transport, ultimately improving gas-sensing performance. This study provides a novel design strategy for developing high-performance room-temperature NO_2_ sensors.

## 1. Introduction

Nitrogen dioxide (NO_2_) is a typical highly oxidizing and toxic gas, primarily emitted from vehicle exhaust, fossil fuel combustion, and industrial activities [1,2]. The U.S. Occupational Safety and Health Administration (OSHA) sets a mandatory ceiling limit of 5 ppm for nitrogen dioxide with no permitted short-term peak exposure. Even at low concentrations, NO_2_ can cause significant irritation and damage to the human respiratory system, making the development of highly sensitive, rapid, and high-selectivity NO_2_-detection technologies highly important [3,4]. Metal oxide semiconductor (MOS) gas sensors have attracted extensive attention in the field of hazardous-gas detection owing to their simple fabrication, low cost, and ease of integration [5]. However, pristine MOS materials generally suffer from limited response at room temperature, slow recovery kinetics, and poor selectivity [6]. Moreover, they often require elevated operating temperatures to achieve satisfactory sensing performance, which increases power consumption and restricts their applications in flexible, portable, and energy-efficient devices [7]. Constructing heterojunctions, such as p–n, p–p, and n–n heterojunctions, through the integration of two MOS materials has been widely recognized as an effective strategy for enhancing sensor response, improving selectivity, and reducing power consumption.

Both ZnO and SnO_2_ are widely used n-type metal oxide semiconductors. ZnO possesses excellent chemical stability and abundant surface-active sites, whereas SnO_2_ exhibits high intrinsic conductivity and rapid electron-transport capability [8,9]. Consequently, the construction of a ZnO/SnO_2_ n–n heterojunction has emerged as a promising approach for NO_2_-sensing applications [10,11]. To date, research on a ZnO/SnO_2_ n–n heterojunction has mainly focused on compositional optimization, morphology engineering, and surface modification, while the influence of different dominant phases on gas-sensing behavior has received comparatively limited attention [12]. ZnO-based sensors modified with SnO_2_ have been extensively investigated. Shah et al. [13] constructed ZnO/SnO_2_ heterojunctions by decorating ZnO nanorods with SnO_2_ nanoparticles, achieving a response of 249.9 toward 5 ppm NO_2_, along with rapid response/recovery characteristics and excellent stability. The sensing performance was significantly superior to that of pristine ZnO. Lou et al. [14] further demonstrated that SnO_2_ decoration markedly enhanced the NO_2_-sensing performance of ZnO nanowires, with the improvement primarily attributed to the charge modulation effect induced by the heterojunction interface. Similarly, employing SnO_2_ as the dominant phase and ZnO as the secondary phase can also effectively improve gas-sensing performance. Ren et al. [15] reported that a ZnO/SnO_2_ heterojunction material exhibited a response of approximately 37 toward 0.2 ppm NO_2_, several times higher than that of pure SnO_2_, while also delivering a lower detection limit and faster response/recovery characteristics. Likewise, the SnO_2_/ZnO composite structure developed by Karaduman et al. [16] enhanced NO_2_ adsorption and facilitated charge transfer, resulting in a synergistic improvement in sensing performance.

The construction of a ZnO/SnO_2_ n-n heterojunction offers several advantages. On the one hand, the heterointerface induces electron redistribution and band bending, thereby significantly amplifying the resistance variation triggered by gas adsorption [17]. On the other hand, the compositing process can improve particle dispersion and optimize the pore structure, leading to an increased number of active sites and enhanced gas diffusion efficiency [18]. Most previous investigations related to ZnO/SnO_2_ heterojunctions primarily concentrated on morphological engineering and simple component modification, generally exploring only one composite configuration where one material acts as the dominant matrix and the other as a minor modifier. Systematic comparative studies on two phase-inverted systems, in which ZnO and SnO_2_ alternately serve as the continuous dominant phase, are still scarce. Herein, both SZ (ZnO-dominant with SnO_2_ modification) and ZS (SnO_2_-dominant with ZnO modification) composites were synthesized using the identical solid-state grinding strategy. We demonstrate that sensing performance cannot be simply positively correlated with oxygen-vacancy concentration. Although SZ-7 exhibits higher oxygen-vacancy content, ZS-7 with SnO_2_ as the dominant phase achieves superior NO_2_-sensing response benefiting from its optimized porous microstructure, efficient charge-transport framework, strengthened NO_2_ adsorption and improved interfacial electronic coupling, which are further verified by DFT calculations. Motivated by these considerations, two phase-inverted ZnO/SnO_2_ sensor systems were synthesized via a solid-state grinding method, with either ZnO or SnO_2_ serving as the dominant phase. The differences in crystal structure, microstructure, surface defects, and pore characteristics between the two systems were systematically investigated. Furthermore, their NO_2_-sensing performances were evaluated at room temperature under 254 nm UV irradiation, with particular emphasis on elucidating the effect of different dominant phases on the sensing behavior. This work provides experimental insights for the rational design of high-performance, highly selective NO_2_-sensing materials.

## 2. Materials and Methods

### 2.1. Chemicals and Reagents

All chemicals used in this study were of analytical reagent (AR) grade and were used as received without any further purification. The reagents included zinc acetate dihydrate (Zn(C_2_H_3_O_2_)_2_·2H_2_O) supplied by Kemiou Chemical Reagent Co., Ltd., Tianjin, China, tin(IV) chloride pentahydrate (SnCl_4_·5H_2_O) purchased from Shanghai Chemical Reagent Procurement and Supply Station, China, cetyltrimethylammonium bromide (CTAB) provided by Bide Pharmatech Co., Ltd., Shanghai, China and potassium hydroxide (KOH) supplied by Baishi Chemical Co., Ltd., Tianjin, China.

### 2.2. Material Preparation

(1) Pure ZnO (Sample Z)

A total of 3 g of zinc acetate dihydrate (Zn(C_2_H_3_O_2_)_2_·2H_2_O) and 0.75 g of cetyltrimethylammonium bromide (CTAB, C_19_H_42_BrN) were thoroughly mixed in a ceramic mortar and ground for 10 min. Subsequently, 1.6 g of KOH was added, and the mixture was further ground until a homogeneous paste was formed. The resulting product was dispersed in deionized water by ultrasonication and repeatedly washed to remove residual ions. The washed sample was then dried at 60 °C for 12 h, followed by grinding into a fine powder and calcination at 300 °C for 3 h. The obtained ZnO powder was denoted as Z.

(2) Pure SnO_2_ (Sample S)

A total of 3 g of tin(IV) chloride pentahydrate (SnCl_4_·5H_2_O) and 0.75 g of CTAB were placed in a ceramic mortar and ground for 10 min. Subsequently, 2 g of KOH was added, and the mixture was further ground until a homogeneous paste was obtained. The resulting product was ultrasonically dispersed in deionized water and repeatedly washed to remove residual ions. After washing, the sample was dried at 60 °C for 12 h, ground into a fine powder, and calcined at 300 °C for 3 h. The obtained SnO_2_ powder was designated as S.

(3) Preparation of ZnO-Dominant SnO_2_-Modified Composites (SZ Series)

A total of 3 g of Zn(C_2_H_3_O_2_)_2_·2H_2_O and 0.75 g of CTAB were weighed, followed by the addition of 0.078 g, 0.18 g, and 0.28 g of SnCl_4_·5H_2_O, corresponding to SnO_2_ mass fractions of 3%, 7%, and 11%, respectively. The mixtures were thoroughly ground in a ceramic mortar for 10 min. Subsequently, 2 g of KOH was added, and grinding was continued until a homogeneous paste was formed. The resulting products were ultrasonically dispersed in deionized water and repeatedly washed to remove residual ions. After washing, the samples were dried at 60 °C for 12 h, ground into fine powders, and calcined at 300 °C for 3 h. The obtained SnO_2_/ZnO composite oxides were denoted as SZ-3, SZ-7, and SZ-11, respectively.

(4) Preparation of SnO_2_-Dominant ZnO-Modified Composites (ZS Series)

A total of 3 g of SnCl_4_·5H_2_O and 0.75 g of CTAB were weighed, followed by the addition of 0.104 g, 0.243 g, and 0.382 g of Zn(C_2_H_3_O_2_)_2_·2H_2_O, corresponding to ZnO mass fractions of 3%, 7%, and 11%, respectively. The mixtures were thoroughly ground in a ceramic mortar for approximately 10 min. Subsequently, 2.2 g of KOH was added, and grinding was continued until a homogeneous paste was formed. The resulting products were ultrasonically dispersed in deionized water and repeatedly washed with deionized water to remove residual ions. After washing, the samples were dried at 60 °C for 12 h, ground into fine powders, and calcined at 300 °C for 3 h. The obtained ZnO/SnO_2_ composite oxides were designated as ZS-3, ZS-7, and ZS-11, respectively.

These mass fractions were selected according to our preliminary experiments, in which the composite with 7 wt% secondary phase delivered the optimal NO_2_-sensing response.

### 2.3. Material Characterization

The phase composition and crystal structure of the samples were characterized using an X-ray diffractometer (XRD, XD-3, Purkinje General Instrument Co., Ltd., Beijing, China) equipped with Cu Kα radiation (λ = 1.5418 Å). The diffraction patterns were recorded over a 2*θ* range of 10°~80° at a scanning rate of 4° min^−1^. The surface morphology of the samples was examined using a scanning electron microscope (SEM, ZEISS Sigma 300, Jena, Germany), and the elemental composition and spatial distribution were analyzed by energy-dispersive X-ray spectroscopy (EDS) attached to the SEM system. The surface chemical composition and valence states of the elements were investigated by X-ray photoelectron spectroscopy (XPS, K-Alpha, Thermo Scientific, East Grinstead, UK). Transmission electron microscopy (TEM) and high-resolution transmission electron microscopy (HRTEM) analyses were performed on a JEM-F200 microscope (JEOL, Akishima-Tokyo, Japan) operated at an accelerating voltage of 200 kV to examine the microstructure and lattice characteristics of the materials. The specific surface area, pore volume, and pore size distribution were determined using an automated surface area and porosity analyzer (BET, TStar II 3020 HD, Micromeritics, Norcross, GA, USA).

### 2.4. Fabrication of Gas Sensors

The gas sensors were fabricated according to the procedure reported in the literature [19]. First, a polyimide (PI) film (thickness: 0.1 mm, Shenzhen Haozhanli Adhesive Technology Co., Ltd., Shenzhen, China) was attached to the surface of a ceramic heating substrate with dimensions of 20 × 20 mm. Subsequently, interdigital electrodes with an active area of 17 × 17 mm were patterned on the PI film using a laser engraving system (DJ 6, Dongguan Diaojiang Intelligent Technology Co., Ltd., Dongguan, China) operated at a laser wavelength of 450 nm and an output power of 1.5 W, with an engraving speed of 15 mm min^−1^. The interdigital electrode consisted of five finger pairs, with a finger length of 8.5 mm and an inter-finger spacing of 0.5 mm.

The as-prepared sensing materials were dispersed in deionized water at a concentration of 15 mg mL^−1^ and ultrasonically treated to obtain a homogeneous suspension. Subsequently, 0.05 mL of the suspension was uniformly drop-cast onto the surface of the interdigital electrodes. After natural drying at room temperature, the devices were aged in an oven at 180 °C for 12 h to improve stability. The resulting sensors were denoted as the SZ-series and ZS-series gas sensors, respectively. The fabrication process of the gas sensors is schematically illustrated in Figure 1.

### 2.5. Gas-Sensing Measurement Procedure

A homemade static gas-sensing measurement system was employed to evaluate the sensing performance of the fabricated sensors, and its schematic illustration is shown in Figure 2. The system mainly consists of a cylindrical transparent test chamber, a UV light source, a signal acquisition unit, and a gas exchange module. The cylindrical test chamber has an inner diameter of 40 mm and a total effective internal volume of 200 mL, and it can be fully sealed using two threaded end caps equipped with sealing rings. A miniature UV lamp (UD04-045LH, Chongqing Haobo Environmental Protection Technology Co., Ltd., Chongqing, China) with a power of 1 W and a wavelength of 254 nm was fixed inside the chamber to provide UV irradiation for sensor activation. The distance between the ultraviolet lamp and the sensor was 1 cm. The sensor resistance was monitored in real time using a digital multimeter (Fluke 287, Fluke Corporation, Everett, WA, USA). For each sensing test, the chamber was sealed, and NO_2_ gas was injected by syringe; the sensor response was recorded under static atmosphere. Gas exchange within the chamber after each measurement cycle was realized by a miniature negative-pressure vacuum pump (Model 555, Fujian Zhuge Liang Electronics Co., Ltd., China). During this post-measurement purge procedure, residual gas inside the chamber was pumped out while fresh dry air was introduced at a constant flow rate of 3 L·min^−1^, so as to remove residual NO_2_ and restore the sensor to its initial baseline resistance.

During the gas-sensing measurements, the sensor was first exposed to ambient air under UV irradiation until a stable resistance was achieved, and the corresponding resistance was recorded as *R*_a_. The target gas was then introduced into the test chamber, and the stabilized resistance was recorded as *R*_g_. The sensor response was defined as the ratio of the resistance in the target gas to that in air (*R*_g_/*R*_a_). The response and recovery times were defined as the time required for the sensor resistance to reach 90% of the total resistance change upon exposure to and removal of the target gas, respectively. Unless otherwise specified, all measurements were conducted at room temperature (25 °C) with a relative humidity of approximately 20% RH. The preparation of NO_2_ gas was obtained by Equation (1):

(1) $$\rho \;(\mathrm{mg}/m^{3})=\frac{M}{22.4}\cdot ppm\cdot \frac{273}{273+T}\cdot \frac{P}{101,325}$$

where *M* is the molecular weight of the gas in g/mol, *P* is the gas pressure in Pa, *T* is the gas temperature in °C, and ppm is the volume in ppm concentration in mL·m^−3^.

In addition, the influence of ambient humidity on the sensing performance was investigated under different relative humidity conditions. For these measurements, the cylindrical test chamber was placed inside a sealed enclosure with a volume of 1 m^3^. During humidity regulation, the chamber remained open, while the ambient humidity was adjusted using an air humidifier (SUPOR, EHTN-06A-300, Shaoxing, China) and continuously monitored with a digital hygrometer (Metrological, JR900, Guangzhou, China). After the humidity stabilized at the desired level, the test chamber was sealed, and the target gas was introduced for gas-sensing measurements.

## 3. Results

### 3.1. Characterization

The XRD patterns of all samples are presented in Figure 3. The diffraction peaks of ZnO can be indexed to the hexagonal wurtzite structure of ZnO (PDF#36-1451). The characteristic peaks located at 31.7°, 34.4°, 36.2°, 47.54°, 56.72°, 62.88°, 66.34°, 67.92°, 69.1°, 72.56°, and 76.9° correspond to the (100), (002), (101), (102), (110), (103), (200), (112), (201), (004), and (202) crystal planes, respectively. The diffraction peaks of SnO_2_ can be assigned to the tetragonal rutile structure of SnO_2_ (PDF#41-1445), with characteristic reflections at 26.6°, 33.9°, 37.5°, 51.8°, 57.88°, 62.26°, 65.44°, and 71.36°, corresponding to the (110), (101), (200), (211), (220), (112), (301), and (202) planes, respectively. For both the ZS and SZ series, no noticeable shift in the diffraction peak positions of ZnO or SnO_2_ was observed after compositing, indicating that both components retained their original crystal structures. With increasing content of the secondary phase, the characteristic diffraction peaks of the dominant phase gradually decreased in intensity, suggesting that the crystallization behavior was influenced by heterojunction formation. This phenomenon is attributed to the formed ZnO-SnO_2_ heterointerfaces. Such grain boundaries act as physical barriers to block grain migration and coalescence of the main ZnO phase, restraining its overgrowth and reconstructing the loose mesoporous composite microstructure [15].

The SEM images of SZ-7 and ZS-7 are shown in Figure 4. Both samples are composed of irregularly shaped nanoparticles with a certain degree of agglomeration and exhibit a loose, porous architecture at different magnifications. Although the overall morphologies of the two composites are similar within the observed scale range, ZS-7 possesses smaller and more uniformly distributed nanoparticles compared with SZ-7. The EDS elemental mapping results reveal that Zn, Sn, and O are homogeneously distributed throughout both samples, with no obvious elemental segregation observed. In ZS-7, the distribution of Sn is dominant, whereas Zn is dominant in SZ-7, consistent with their respective dominant-phase compositions. These results confirm the formation of uniformly dispersed ZnO/SnO_2_ n-n heterojunction systems in both composites. Combined analysis of the morphological and elemental distribution characteristics indicates that the finer and more homogeneous particle structure of ZS-7 is favorable for providing more exposed surface-active sites. Meanwhile, the uniformly distributed heterointerfaces can provide additional active adsorption sites and facilitate gas diffusion, adsorption, and interfacial reactions [17]. These structural advantages establish a solid foundation for the superior gas-sensing performance of ZS-7.

The HRTEM images of SZ-7 and ZS-7 are presented in Figure 5. The interconnected stacking of nanoparticles within the composites is conducive to the formation of continuous charge-transport pathways, thereby providing an effective structural framework for electrical signal conduction during gas-sensing measurements [20]. For SZ-7, lattice fringes with interplanar spacings of 0.335 nm and 0.281 nm can be assigned to the (110) plane of SnO_2_ and the (100) plane of ZnO, respectively. The same characteristic lattice planes are also observed in ZS-7, confirming the coexistence of ZnO and SnO_2_ crystalline phases and the successful formation of ZnO/SnO_2_ heterojunctions while preserving the intrinsic crystal structures of both components. The formation of these heterojunctions is beneficial for interfacial charge redistribution and carrier-transport regulation, providing a microstructural basis for the modulation of interfacial potential barriers and variations in the electron depletion layer during the NO_2_-sensing process.

As shown in Figure 6a,b, only the characteristic Zn 2p and O 1s peaks are observed in the XPS survey spectrum of pure ZnO (Z), whereas only the Sn 3d and O 1s peaks appear in that of pure SnO_2_ (S). In contrast, both Zn 2p and Sn 3d signals are detected in the survey spectra of ZS-7 and SZ-7, confirming the successful incorporation of the secondary phase into the dominant phase matrix. This result provides direct evidence for the formation of interfacial contact and ZnO/SnO_2_ heterojunctions within the composites. The high-resolution Zn 2p spectra of Z, ZS-7, and SZ-7 are shown in Figure 6c,d. The characteristic peaks located at 1021.6 eV and 1044.7 eV correspond to Zn 2p_3/2_ and Zn 2p_1/2_, respectively, indicating that Zn predominantly exists in the Zn2^+^ oxidation state. The high-resolution Sn 3d spectra of S, ZS-7, and SZ-7 are presented in Figure 6e,f. For pure SnO_2_, the Sn 3d_5/2_ and Sn 3d_3/2_ peaks are located at 486.1 eV and 494.5 eV, respectively. After heterojunction formation, both peaks shift toward higher binding energies. Specifically, the Sn 3d_5/2_ and Sn 3d_3/2_ peaks are located at 486.8 eV and 495.2 eV for ZS-7, and at 486.6 eV and 495.1 eV for SZ-7. The positive shift in the Sn 3d binding energies originates from electron transfer from SnO_2_ to ZnO across the n–n heterojunction, which modifies the electronic environment of SnO_2_ and induces interfacial electron redistribution. Such electronic modulation accelerates charge transport and strengthens the interaction between the material and NO_2_ molecules, thereby delivering superior sensing performance of ZS-7.

Figure 7a–d presents the deconvoluted O 1s XPS spectra of Z, S, SZ-7 and ZS-7. The resolved peaks can be assigned to lattice oxygen (O_L_), oxygen vacancies (O_V_) and chemisorbed oxygen (O_C_), respectively [21]. The characteristic peaks belonging to O-H and C-O bonds are too weak and overlap with the chemisorbed oxygen component, so they cannot be separated into individual fitting peaks. The relative contents of the three oxygen species are summarized in Table 1 As critical active sites for NO_2_ adsorption and electron transfer, appropriate oxygen vacancy content facilitates the adsorption and activation of gas molecules [22]. The relative O_V_ content of SZ-7 is 26.28%, which is obviously higher than the value of 16.50% for ZS-7. Although SZ-7 possesses a higher oxygen vacancy concentration, it exhibits inferior gas-sensing performance compared with ZS-7.

Figure 8 presents the N_2_ adsorption–desorption isotherms of Z, S, SZ-7, and ZS-7, while the corresponding BET parameters are summarized in Table S1. All samples exhibit pronounced hysteresis loops in the medium-to-high relative pressure region (P/P_0_ > 0.4) and display typical type-IV isotherms, indicating the presence of predominantly mesoporous structures [23]. Pure ZnO exhibits a specific surface area of 20.1 m^2^·g^−1^ and a pore volume of 0.074 cm^3^·g^−1^. After SnO_2_ modification, SZ-7 shows an increased specific surface area (47.5 m^2^·g^−1^) and pore volume (0.144 cm^3^·g^−1^). This enhancement can be attributed to the suppression of ZnO particle agglomeration by SnO_2_, which facilitates the formation of a larger accessible surface area. Meanwhile, partial blockage of ZnO nanopores by the introduced SnO_2_ leads to a slight decrease in pore size. Pure SnO_2_ possesses a remarkably high specific surface area of 145.82 m^2^·g^−1^, but its average pore diameter is only 3 nm. Such small pores can hinder the diffusion and desorption of NO_2_ molecules within the pore channels, preventing the high surface area from being fully translated into effective sensing performance [24]. For ZS-7, the specific surface area decreases to 70.30 m^2^·g^−1^ after ZnO modification. Nevertheless, ZS-7 exhibits a relatively large pore volume and pore diameter, achieving a more favorable balance between the availability of adsorption sites and gas diffusion kinetics. This optimized porous architecture provides efficient pathways for NO_2_ adsorption and mass transport [24,25], thereby contributing to its superior gas-sensing performance.

### 3.2. Gas-Sensing Performance of the Sensors

Figure S1a,b show the resistance variation curves of the SZ-7 and ZS-7 sensors under alternating UV-on and UV-off conditions. Under UV off conditions, the baseline resistance of SZ-7 reaches approximately 570 MΩ; after turning on 254 nm UV irradiation, its resistance sharply drops down to roughly 15 MΩ. For ZS-7, its resistance decreases from ~14 MΩ (UV-off) to ~1.8 MΩ under UV-on conditions, demonstrating that both composites exhibit pronounced photoresponse behavior. Upon irradiation with 254 nm UV light, photogenerated electron–hole pairs are produced within the sensing materials. The photogenerated holes react with surface-adsorbed oxygen species, promoting their desorption and releasing trapped electrons back into the conduction band. As a result, the thickness of the surface electron depletion layer decreases, leading to a rapid reduction in resistance until a stable UV-induced baseline resistance is reached [26]. After the UV light is switched off, oxygen molecules are re-adsorbed onto the surface and capture electrons, causing the sensor resistance to increase gradually. Notably, SZ-7 exhibited a much larger resistance modulation than ZS-7, whereas ZS-7 maintained a consistently lower resistance level throughout the measurement, indicating more efficient charge transport. In addition, the resistance recovery process of ZS-7 after UV removal is relatively slow, suggesting a lower recombination rate of photogenerated carriers and a higher carrier separation efficiency under UV excitation. The prolonged carrier lifetime helps sustain active surface reactions, thereby facilitating the adsorption and reaction of gas molecules and ultimately contributing to the enhanced gas-sensing performance of ZS-7.

Figure S2a presents the baseline resistances of all sensors under continuous irradiation with 254 nm UV light. The baseline resistance of the pure ZnO sensor was lower than that of pure SnO_2_, indicating that ZnO exhibits higher conductivity under UV activation. Among the composite sensors, the ZS series generally showed lower baseline resistances than the SZ series, while SZ-11 exhibits the highest baseline resistance. These results suggest that using SnO_2_ as the dominant phase is more favorable for establishing continuous electron-transport pathways, consistent with the structural and morphological analyses discussed above. The dynamic resistance response curves of the sensors toward different concentrations of NO_2_ are shown in Figure S2b–i. All sensors exhibit pronounced resistance modulation upon exposure to NO_2_, confirming their typical response characteristics toward this oxidizing gas. Under identical NO_2_ concentrations, all composite sensors display larger resistance variations than their corresponding pristine counterparts, indicating that the construction of ZnO/SnO_2_ n-n heterojunction effectively enhances the NO_2_-sensing performance.

Figure 9b–i show the sensor responses toward different concentrations of NO_2_. For all sensors, the response increased progressively with increasing NO_2_ concentration. The responses of all composite materials are higher than those of the corresponding pristine phases. Moreover, the response of the composites initially increases and then decreases with increasing content of the secondary phase, reaching a maximum at a modifier loading of 7 wt%. This result indicates that an appropriate amount of modification is beneficial for enhancing the sensing performance of the heterojunction, whereas excessive modification deteriorates the sensing characteristics. The latter can be attributed to the disruption of the continuity of the dominant phase, which hinders electron transport and reduces conductive connectivity within the sensing network [27]. The linear fitting results between sensor response and NO_2_ concentration are presented in Figure 9a, and the corresponding correlation coefficients (R^2^) and sensitivities (S, defined as the slope of the fitted line) are summarized in Table S2. Figure 9b–i displays the dynamic response curves of all sensors to various concentrations of NO_2_. All sensors exhibit excellent linearity over the tested concentration range, with R^2^ values exceeding 0.98, indicating strong potential for quantitative detection of low-concentration NO_2_. The sensitivities of SZ-7 and ZS-7 are 1.434 ppm^−1^ and 39.446 ppm^−1^, respectively, demonstrating that ZS-7 is substantially more sensitive to NO_2_ than SZ-7. The limit of detection (*LOD*) was calculated from the root mean square (*RMS*) value of the baseline noise and the slope of the calibration curve according to the following Equation (2) [28]:

*LOD* = 3 × *RMS*/*slope* (2)

The calculated *LOD* values were 84 ppb for SZ-7 and 74 ppb for ZS-7, indicating that the ZnO/SnO_2_ n-n heterojunction enables effective detection of low-concentration NO_2_ under UV activation [29]. The average response/recovery times of SZ-7 are 21 s/164 s, whereas those of ZS-7 are 35 s/122 s. In addition to its much higher sensing response, ZS-7 exhibits faster recovery speed, although its response time is longer than that of SZ-7. It achieves a lower detection limit and overall superior sensing capability. Combined with the aforementioned structure and surface analyses, it is noteworthy that although SZ-7 possesses a higher oxygen-vacancy content than ZS-7, the latter delivers a significantly higher sensing response. This finding can be attributed to the larger specific surface area of ZS-7 and the better preservation of conductive pathways provided by its SnO_2_-dominant framework.

As shown in Figure 10a, both SZ-7 and ZS-7 exhibit excellent repeatability toward 9 ppm NO_2_, with nearly unchanged response values over multiple sensing cycles. The response values of SZ-7 and ZS-7 toward various interfering gases at 100 ppm (hydrogen sulfide, ammonia, ethanol, acetone, diethyl ether, acetic acid, ethyl acetate, toluene, isopropanol, and dichloromethane) are summarized in Table S3, and the corresponding selectivity comparison is presented in Figure 10b. For both sensors, the response toward 1 ppm NO_2_ is significantly higher than those toward the interfering gases, demonstrating excellent selectivity. Among the tested sensors, ZS-7 exhibits the highest response, highlighting its superior selectivity for NO_2_ detection. The long-term stability results of SZ-7 and ZS-7 are shown in Figure 10c. Over a testing period of 28 days, both sensors maintain relatively stable responses toward NO_2_ with only minor fluctuations, indicating negligible performance degradation during long-term operation. Figure 10d presents the responses of SZ-7 and ZS-7 toward 9 ppm NO_2_ under different relative humidity (RH) conditions. The response of both sensors gradually decreased with increasing RH, which can be attributed to the competitive adsorption of water molecules on the sensor surface. Adsorbed water occupies active sites and suppresses the effective adsorption of NO_2_ molecules. Nevertheless, ZS-7 retains a higher response than SZ-7 under humid conditions, indicating that the SnO_2_-dominant composite possesses superior moisture tolerance and environmental adaptability. Overall, ZS-7 outperforms SZ-7 in terms of repeatability, selectivity, long-term stability, and humidity resistance, further demonstrating the advantage of employing SnO_2_ as the dominant phase for enhancing the NO_2_-sensing performance of ZnO/SnO_2_ n-n heterojunction composites.

Table 2 compares the NO_2_-sensing performance of the SZ-7 and ZS-7 sensors with those of previously reported SnO_2_/ZnO-based sensors. The comparison revealed that the ZS-7 sensor developed in this work achieved highly sensitive detection of 1 ppm NO_2_ at room temperature under 254 nm UV irradiation, demonstrating its great application potential for NO_2_ monitoring.

### 3.3. Gas-Sensing Mechanism

Figure 11a illustrates the energy band structures of pure ZnO and SnO_2_. The band gaps of ZnO and SnO_2_ are 3.37 eV and 3.60 eV, respectively [34,35]. Figure 11b schematically depicts the band alignment after the formation of a ZnO/SnO_2_ n-n heterojunction. Owing to the higher work function of ZnO relative to SnO_2_, electrons spontaneously transfer from SnO_2_ to ZnO until their Fermi levels reach equilibrium. This interfacial electron redistribution induces band bending and generates a built-in electric field at the interface, thereby establishing an n-n heterojunction [36].

In air, oxygen molecules are first adsorbed onto the surface of the composite materials (Equation (3)) and subsequently capture electrons from the conduction band to form chemisorbed oxygen species, predominantly O_2_^−^ at temperatures below 100 °C (Equation (5)). This process decreases the surface electron concentration, resulting in the formation of an electron depletion layer and an increase in sensor resistance [37]. For the SZ series, where ZnO serves as the continuous dominant phase, oxygen molecules are primarily adsorbed on the ZnO surface and extract electrons from its conduction band, leading to the formation of a relatively wide electron depletion layer on ZnO [26]. In contrast, for the ZS series, where SnO_2_ acts as the continuous dominant phase, oxygen adsorption mainly occurs on the SnO_2_ surface, accompanied by electron extraction from the SnO_2_ conduction band. Although electron transfer from SnO_2_ to ZnO exists at the heterojunction interface, this compensation effect is confined to the local interfacial region. Moreover, because SnO_2_ possesses a higher carrier concentration than ZnO [38], the SZ series exhibits a higher overall resistance state than the ZS series [36], which is consistent with the experimental results discussed above. Upon exposure to NO_2_, the gas molecules further capture electrons from the sensor surface and form adsorbed ionic species (e.g., NO_2_^−^) (Equation (5)), causing further expansion of the surface depletion layer [39]. Simultaneously, electrons in the conduction bands of both SnO_2_ and ZnO near the heterojunction interface are consumed by NO_2_ adsorption, leading to a reduction in interfacial electron concentration. Consequently, the heterojunction barrier height increases, hindering carrier transport across the interface and resulting in a rapid increase in the overall sensor resistance [39].

(3) $$O_{2}\left(\mathrm{gas}\right)\;\to {\;O}_{2}\left(\mathrm{ads}\right)$$

(4) $$O_{2}\left(\mathrm{ads}\right)+e^{-}\;\to \;O_{2}^{-}\left(\mathrm{ads}\right)$$

(5) $${\mathrm{NO}}_{2}+e_{\left(\mathrm{hv}\right)}^{-}\;\to {\;\mathrm{NO}}_{2}^{-}\left(\mathrm{ads}\right)$$

To further elucidate the interaction between NO_2_ molecules and the sensing materials, NO_2_ adsorption models on the surfaces of ZnO, SnO_2_, SZ, and ZS were constructed using the Materials Studio 2020 package based on density functional theory (DFT), as shown in Figure 12a–d. First-principles DFT calculations were carried out via the CASTEP module. The generalized-gradient approximation (GGA) with the Perdew–Burke–Ernzerhof (PBE) exchange-correlation functional was adopted to describe electron exchange-correlation effects, and ultrasoft pseudopotentials were used to represent the interactions between ionic cores and valence electrons. The plane-wave basis-set cutoff energy was set to 540 eV. The Monkhorst-Pack k-point mesh of 1 × 1 × 1 was applied for Brillouin-zone sampling. Geometry optimization was subsequently performed for all models, and the projected density of states (PDOS), as well as the adsorption energies, were calculated to investigate the adsorption behavior and electronic interactions between NO_2_ molecules and the material surfaces. The adsorption energy was calculated according to the following Equation (6) [40]:

(6) $$E_{\mathrm{ads}}=E_{\mathrm{total}}-E_{\mathrm{surface}}-E_{NO_{2}}$$

where $E_{ads}$ is the adsorption energy, $E_{total}$ is the total energy of the adsorption system, $E_{surface}$ is the energy of the clean surface, and $E_{NO_{2}}$ is the energy of an isolated NO_2_ molecule. The calculated adsorption energies were −0.0336 eV for Z, −0.097 eV for SZ, −0.437 eV for ZS, and −0.605 eV for S. In general, a more negative adsorption energy indicates a stronger interaction between NO_2_ molecules and the material surface. Accordingly, the NO_2_ adsorption capability follows the order: S > ZS > SZ > Z. Among the investigated materials, pure SnO_2_ exhibits the strongest adsorption toward NO_2_, whereas pure ZnO shows the weakest adsorption. The adsorption strengths of the composite systems lie between those of the two pristine materials. Notably, the ZS series, in which SnO_2_ serves as the dominant phase, exhibits significantly stronger NO_2_ adsorption than the SZ series, where ZnO is the dominant phase. These results demonstrate that the nature of the dominant phase plays a crucial role in governing the adsorption behavior of NO_2_ on the material surface. The continuous SnO_2_ framework is more favorable for enhancing the capture and adsorption of NO_2_ molecules, which contributes to the improved sensing performance of the ZS-series sensors.

Figure 12e–h present the projected density of states (PDOS) of ZnO, SnO_2_, SZ, and ZS, respectively. For pristine ZnO and SnO_2_, the electronic states are mainly distributed in the lower-energy region, while the density of states near the Fermi level (0 eV) remains relatively low, indicating that the number of electronic states directly available for charge transfer at the material surface is limited [41]. In contrast, the composite systems SZ and ZS exhibit significantly enhanced total density-of-states peaks, particularly within the energy range of −10 to 0 eV. Moreover, the density of states near the Fermi level is markedly higher than that of the pristine materials. Simultaneously, the s, p, and d orbitals display more pronounced overlap in the composite systems, suggesting the occurrence of orbital coupling and interfacial orbital hybridization following the formation of the ZnO/SnO_2_ heterojunction, which gives rise to new electronic states at the interface [42]. This interfacial electronic coupling facilitates the separation and transport of charge carriers and enhances the charge-transfer interaction between the composite surface and NO_2_ molecules. Consequently, the heterojunction structure promotes more efficient electronic communication during the gas-sensing process, contributing to the improved sensing performance of the composite materials.

A further comparison between the two composite systems reveals that the ZS series not only exhibits a more negative adsorption energy than the SZ series, but also shows a more pronounced increase in the density of electronic states near the Fermi level relative to the corresponding pristine materials. These results indicate stronger interfacial electronic coupling and more active charge transport in the ZS composites. Consequently, when NO_2_ molecules are adsorbed onto the ZS surface, electrons can be transferred more readily from the sensing material to the adsorbed NO_2_ species, resulting in a more significant resistance modulation. Combined with the adsorption-energy analysis, it can be inferred that although pure SnO_2_ exhibits the strongest NO_2_ adsorption, excessively strong adsorption may hinder gas desorption and delay sensor recovery. In contrast, the ZS series achieves a favorable balance between adsorption strength and interfacial electronic properties, providing sufficiently strong NO_2_ adsorption while maintaining efficient charge transfer and reversible surface reactions. Therefore, the ZS composites exhibit superior overall sensing performance. This conclusion is in good agreement with the experimental observations, where the ZS-series sensors demonstrate higher responses and lower detection limits toward NO_2_ than the corresponding SZ-series sensors.

In addition to n-n heterojunction engineering, UV-light activation represents another effective strategy for achieving room-temperature gas sensing with metal oxide semiconductors [43]. Under UV irradiation, photogenerated electron–hole pairs are generated within the sensing material, which facilitates the desorption and re-adsorption of surface oxygen species. As a result, the surface reaction barrier is reduced under room-temperature conditions, leading to enhanced responsiveness toward target gas molecules [17]. Compared with conventional thermal activation, the UV-light-assisted approach is more energy efficient and offers a promising route for the development of high-selectivity, flexible gas sensors operating at room temperature [44]. Therefore, the combination of heterojunction modulation and UV-light activation provides a synergistic strategy for realizing highly efficient room-temperature NO_2_ detection using ZnO/SnO_2_-based composite materials.

## 4. Conclusions

In this work, two phase-inverted ZnO/SnO_2_ composite systems were successfully synthesized via a solid-state grinding method: the SZ series, consisting of ZnO as the dominant phase and SnO_2_ as the secondary phase, and the ZS series, consisting of SnO_2_ as the dominant phase and ZnO as the secondary phase. Their room-temperature NO_2_-sensing performances were systematically investigated under 254 nm UV irradiation. The results showed that ZS-7 possessed finer and more uniformly distributed grains, together with a larger specific surface area (70.30 m^2^·g^−1^), which was favorable for the adsorption and diffusion of gas molecules. Although SZ-7 exhibited a higher oxygen-vacancy content (26.28%), the enhancement of sensing performance was not simply proportional to the concentration of oxygen vacancies. Instead, optimal sensing behavior requires a synergistic interplay between oxygen vacancies, the conductivity of the dominant phase, and the heterostructure configuration. Overall, the ZS series, with SnO_2_ as the dominant phase, exhibited superior sensing performance compared with the SZ series. Among all samples, ZS-7 achieved a sensitivity of 39.446, response/recovery times of 35 s/122 s, and a detection limit of 74 ppb, whereas SZ-7 exhibited a sensitivity of 1.434, response/recovery times of 21 s/164 s, and a detection limit of 84 ppb. DFT calculations revealed that the ZS series exhibits stronger NO_2_ adsorption than the SZ series, while the composite materials possess a higher density of electronic states near the Fermi level than the corresponding pristine materials. The formation of ZnO/SnO_2_ n-n heterojunction effectively regulates interfacial charge distribution and promotes the separation and migration of photogenerated charge carriers under UV irradiation, thereby enhancing surface reaction activity. This study provides new insights into the structural design and performance optimization of metal oxide gas-sensing materials and offers valuable guidance for the development of high-performance, high-selectivity gas sensors.

## Figures and Tables

**Figure 1:**
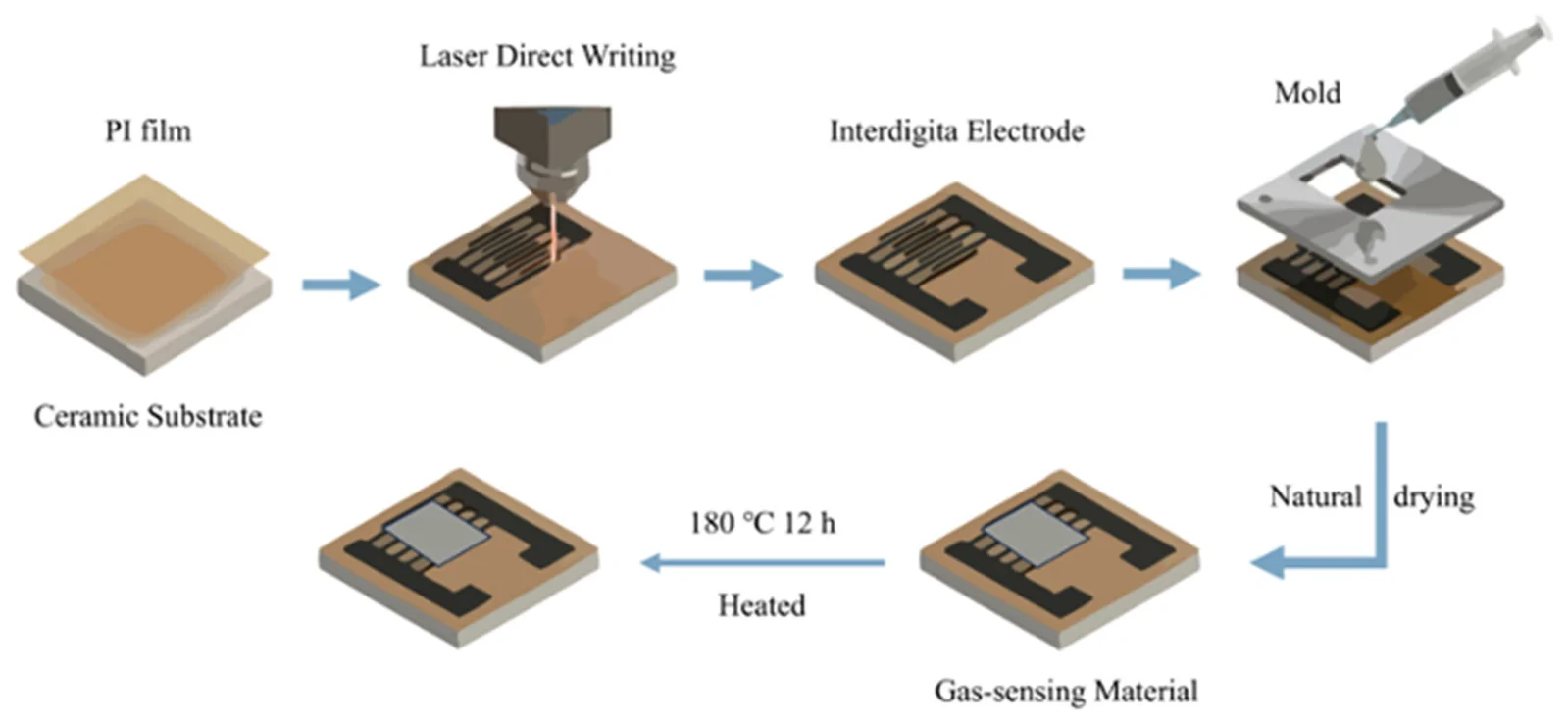
Flow chart of sensor preparation.

**Figure 2:**
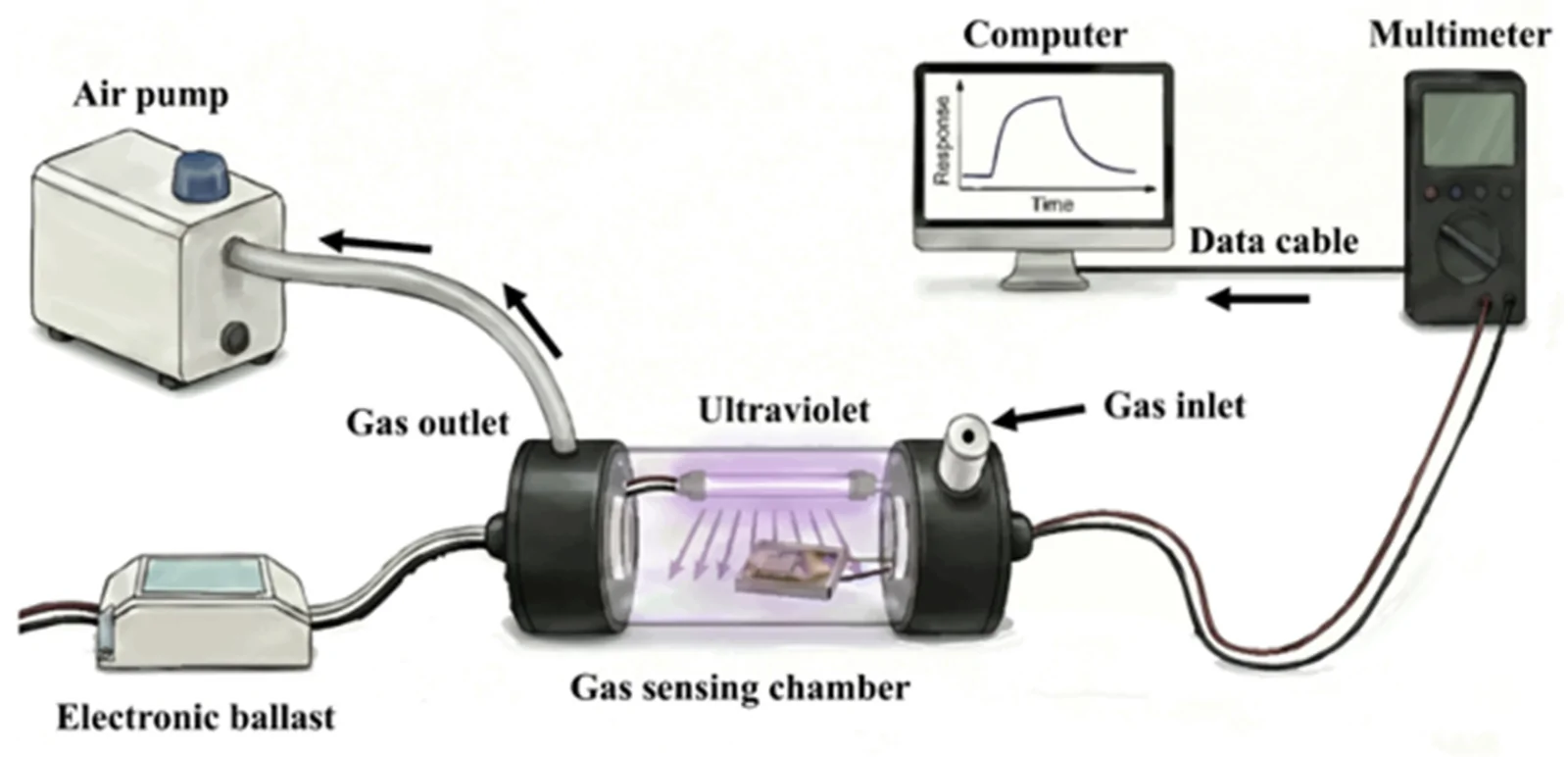
Static gas sensitivity test system.

**Figure 3:**
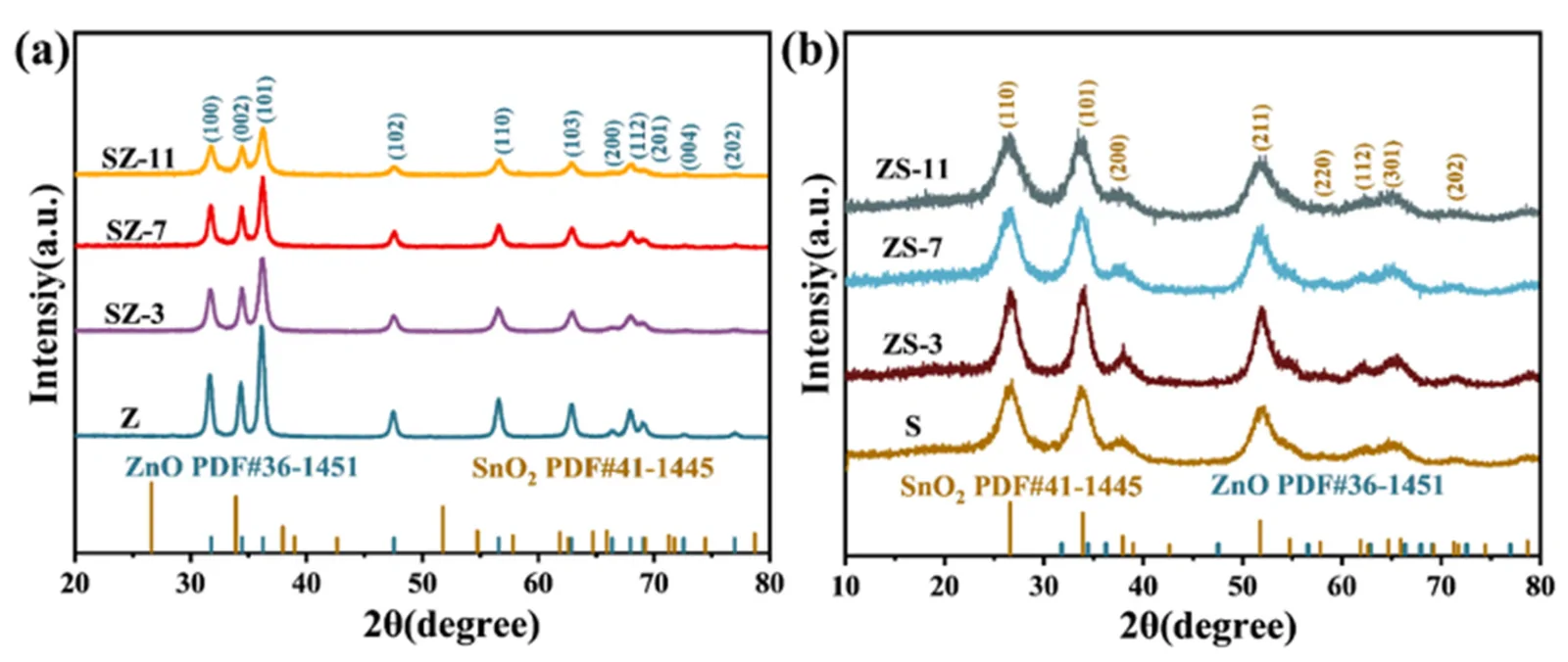
(**a**) XRD patterns of pure ZnO and SZ-3, SZ-7, and SZ-11; (**b**) XRD patterns of pure SnO_2_ and ZS-3, ZS-7, and ZS-11.

**Figure 4:**
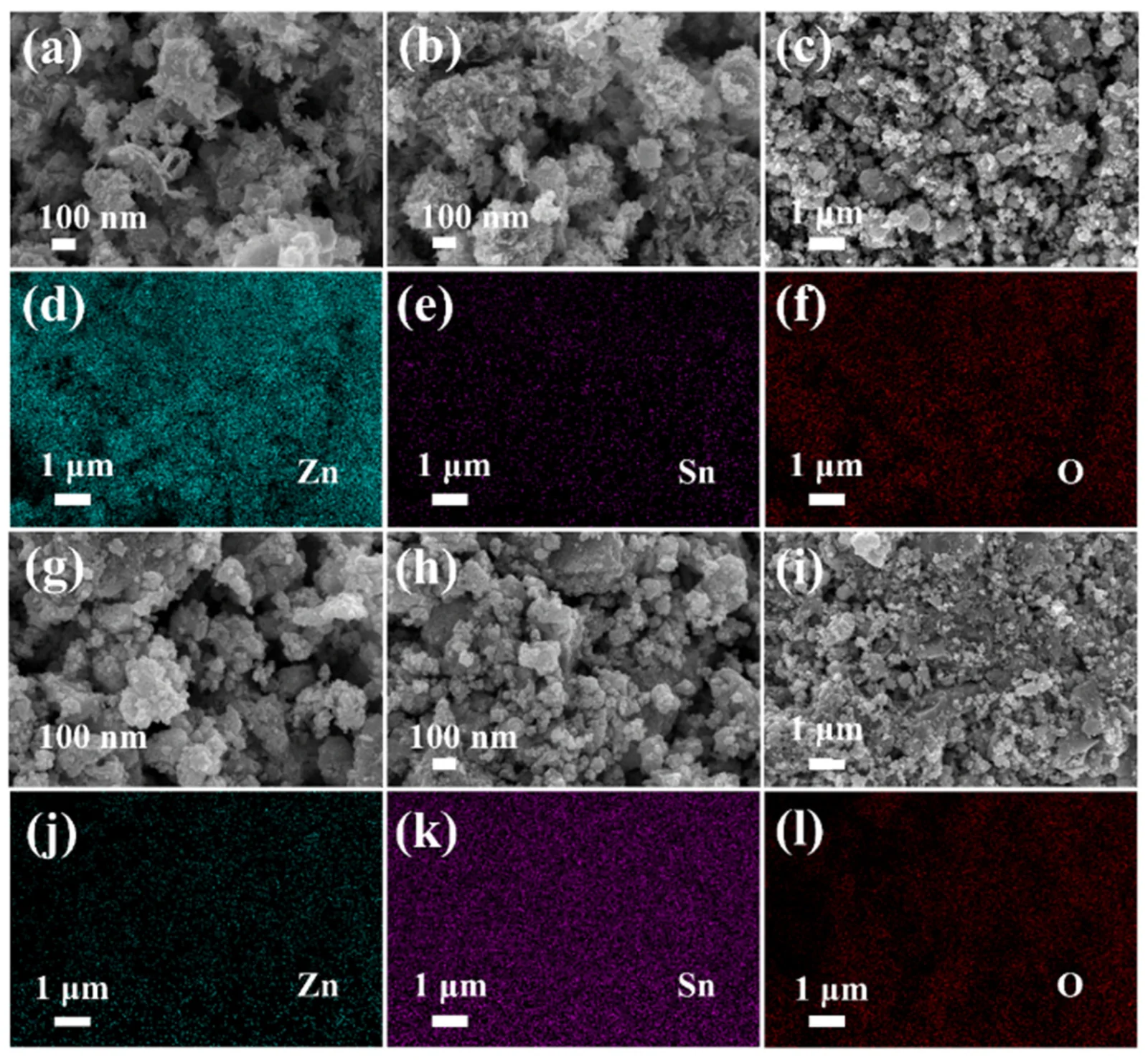
(**a**–**c**) SEM images of SZ-7; (**d**–**f**) corresponding EDS elemental mapping of (**c**); (**g**–**i**) SEM images of ZS-7; and (**j**–**l**) corresponding EDS elemental mapping of (**i**).

**Figure 5:**
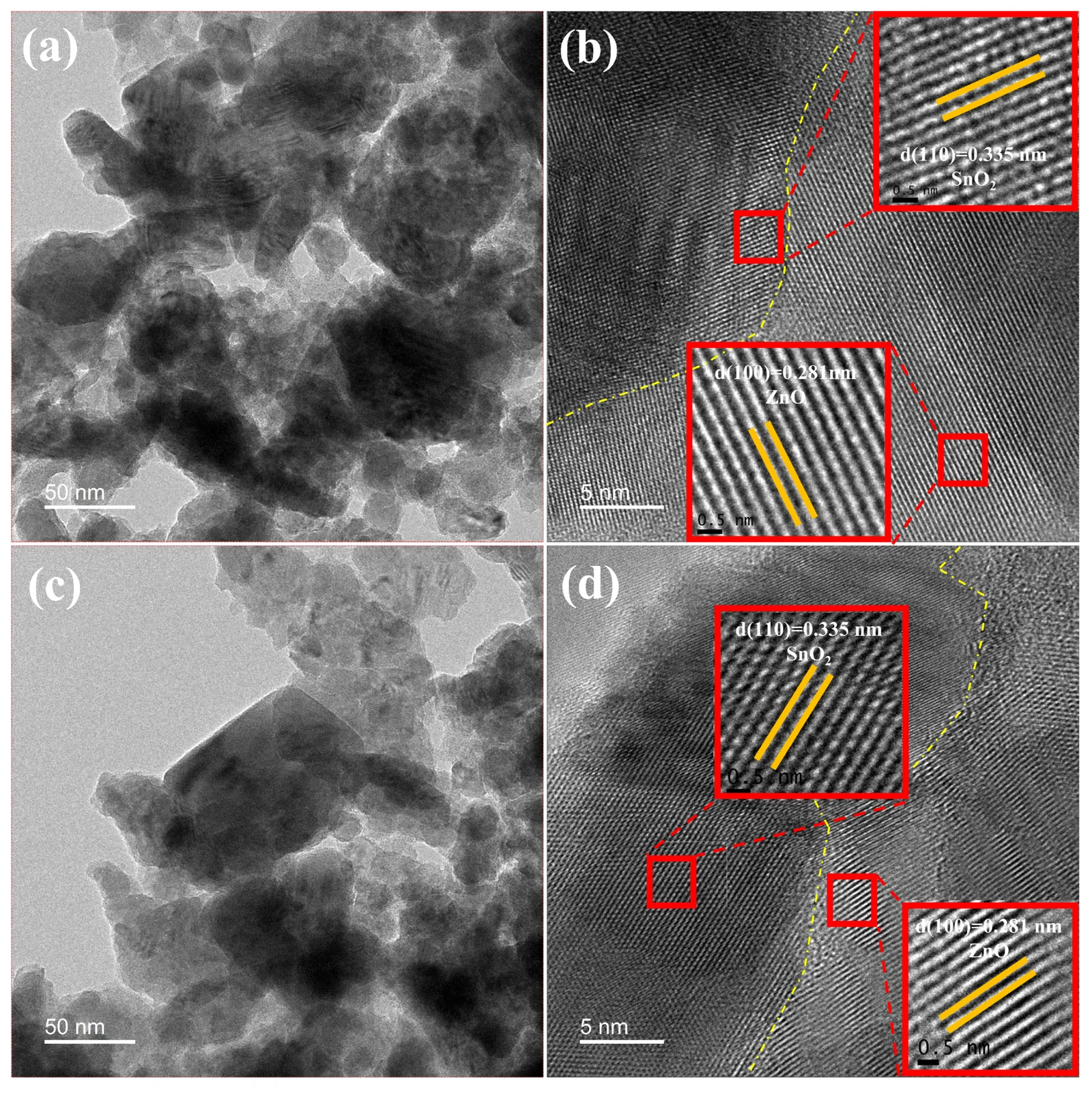
Both (**a**) and (**b**) are the HRTEM images of SZ-7, while (**c**) and (**d**) are the HRTEM images of ZS-7, respectively.

**Figure 6:**
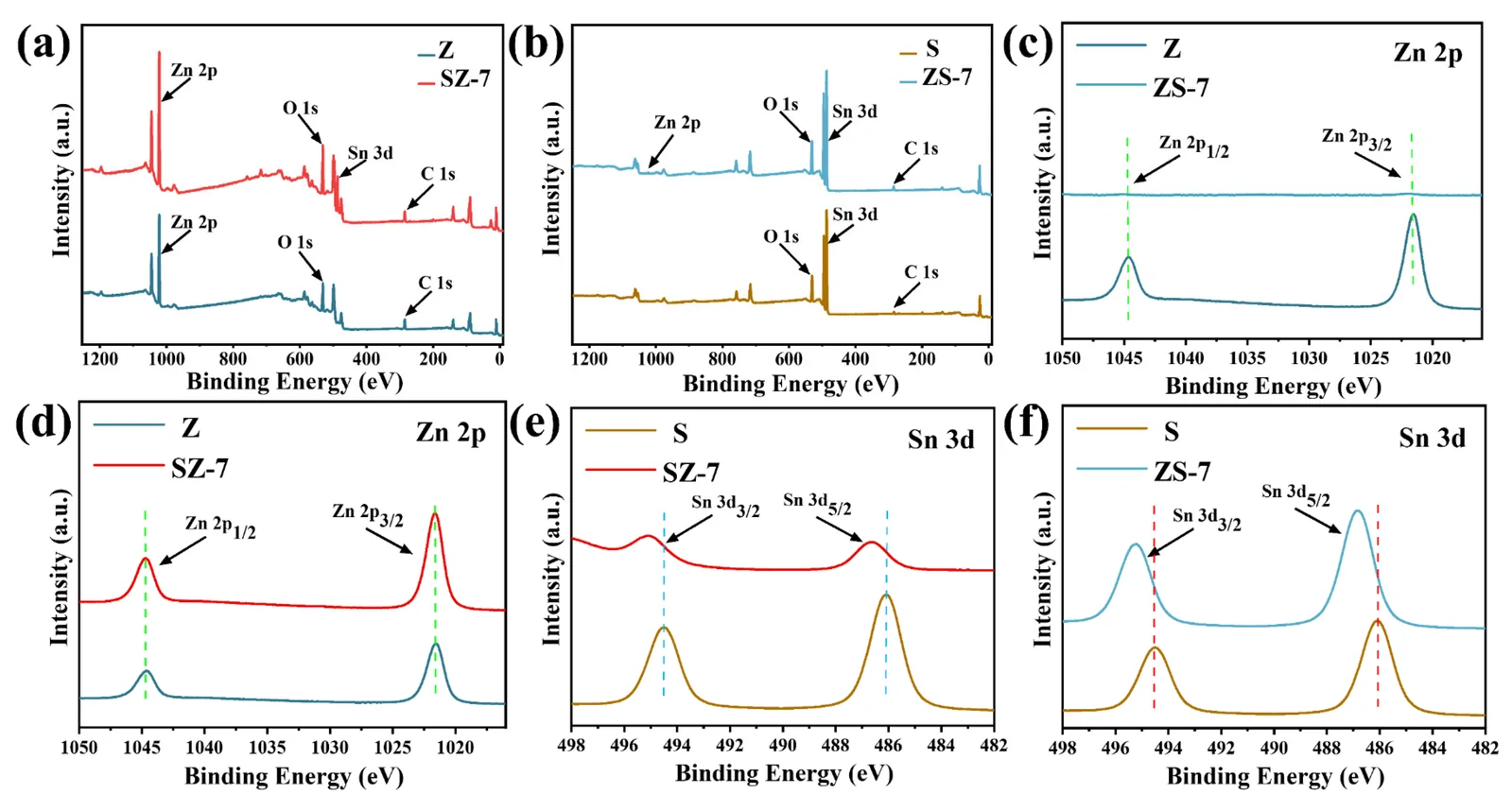
(**a**) XPS survey spectra of Z and SZ-7. (**b**) XPS survey spectra of S and ZS-7. (**c**) High-resolution Zn 2p spectra of Z and ZS-7. (**d**) High-resolution Zn 2p spectra of Z and SZ-7. (**e**) High-resolution Sn 3d spectra of S and ZS-7. (**f**) High-resolution Sn 3d spectra of S and SZ-7.

**Figure 7:**
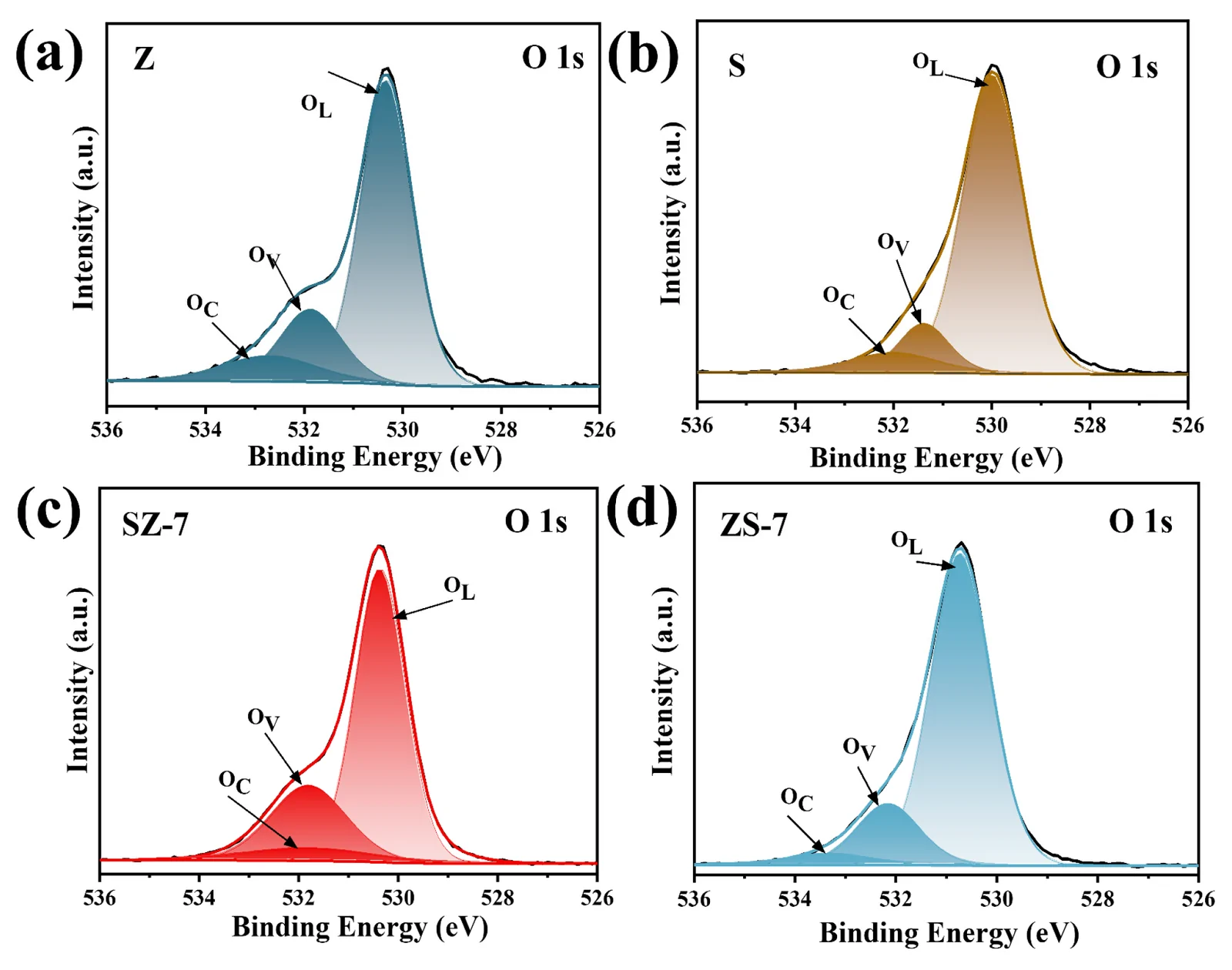
High-resolution O 1s spectra of (**a**) Z, (**b**) S, (**c**) SZ-7, and (**d**) ZS-7.

**Figure 8:**
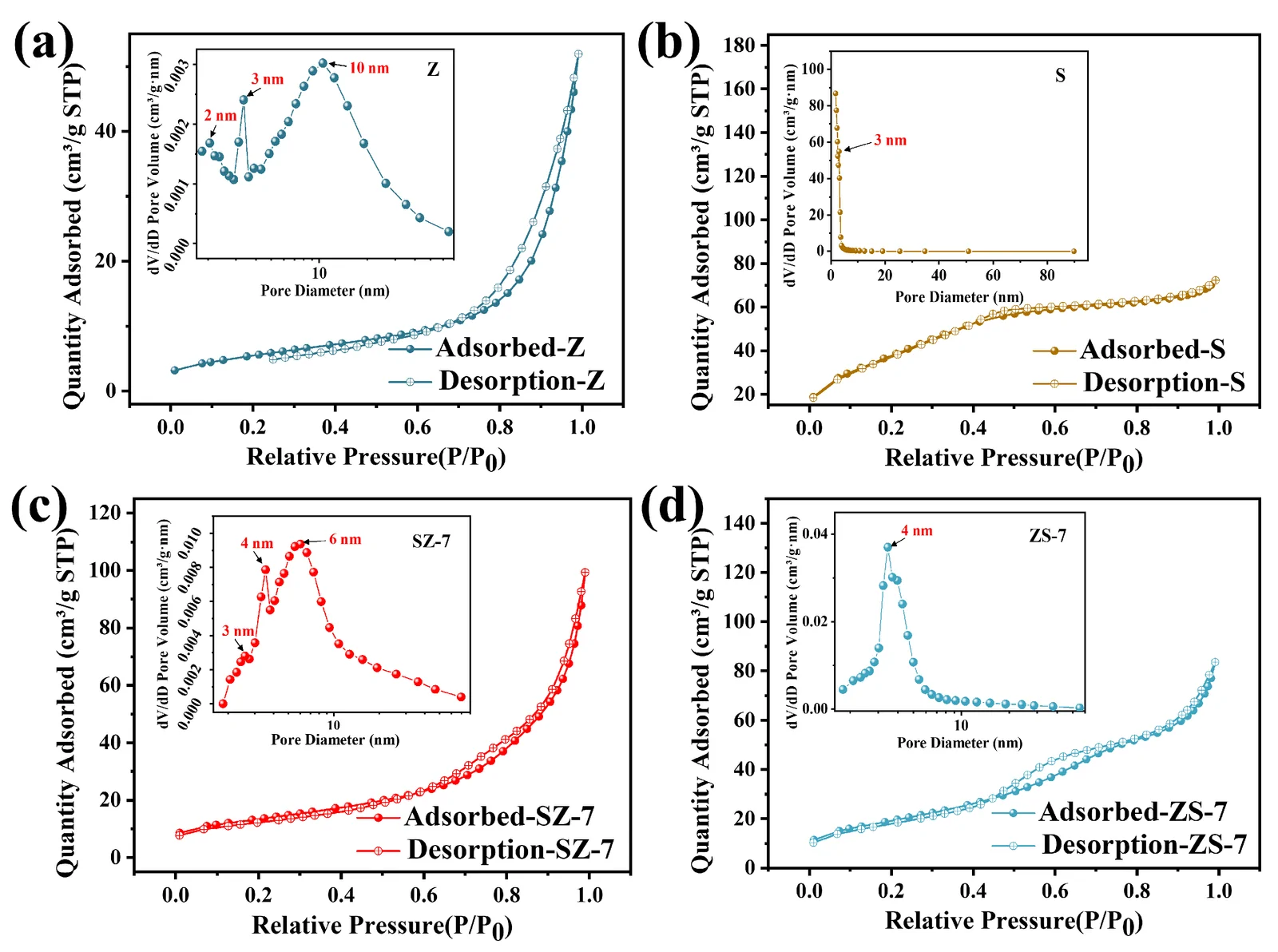
(**a**–**d**) N_2_ adsorption–desorption isotherms and corresponding pore size distribution curves of the Z, S, SZ-7, and ZS-7.

**Figure 9:**
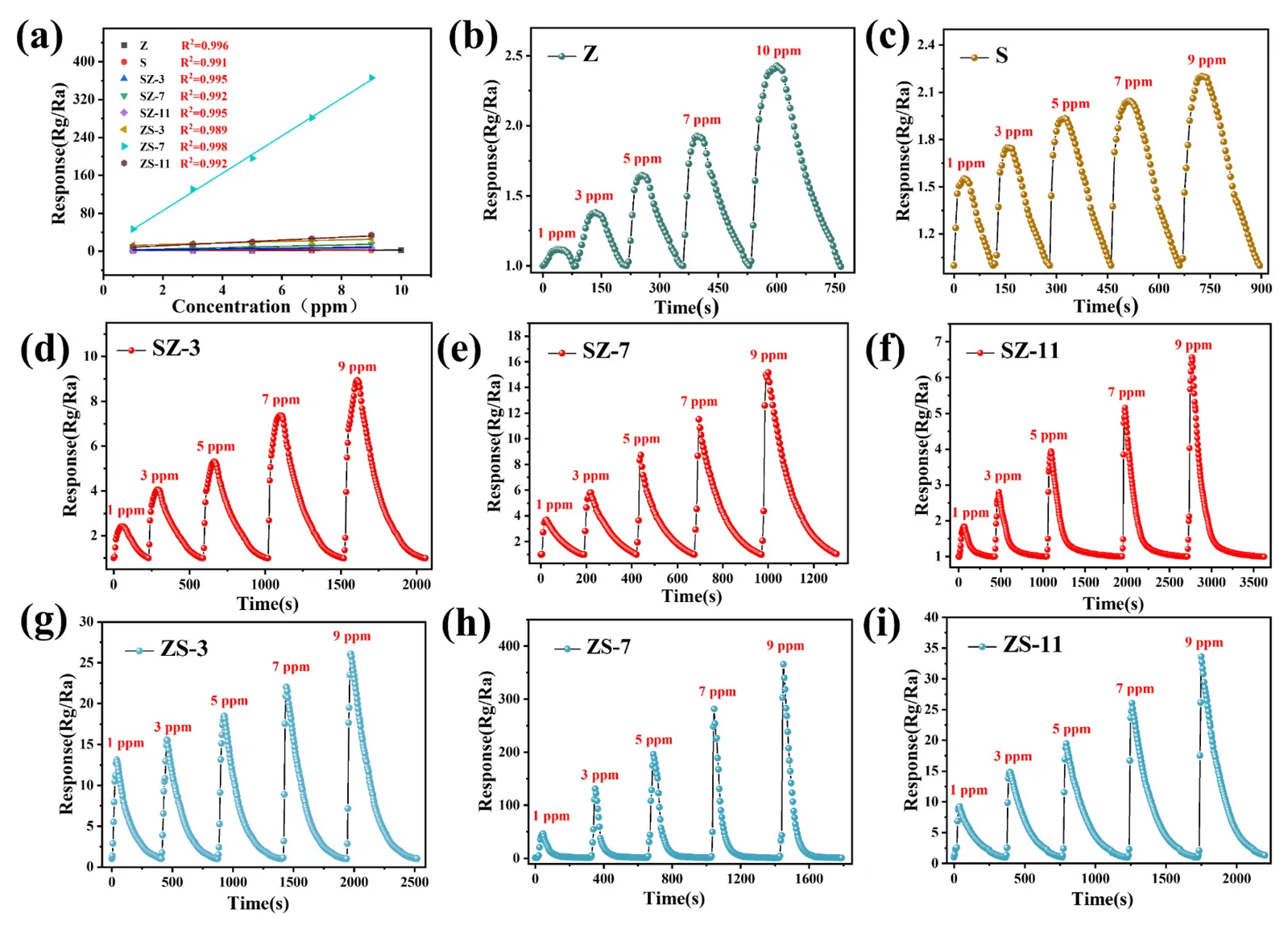
(**a**) Linear fitting curves of response versus NO_2_ concentration for different sensors; (**b**–**i**) the dynamic response curves of all sensors to various concentrations of NO_2_.

**Figure 10:**
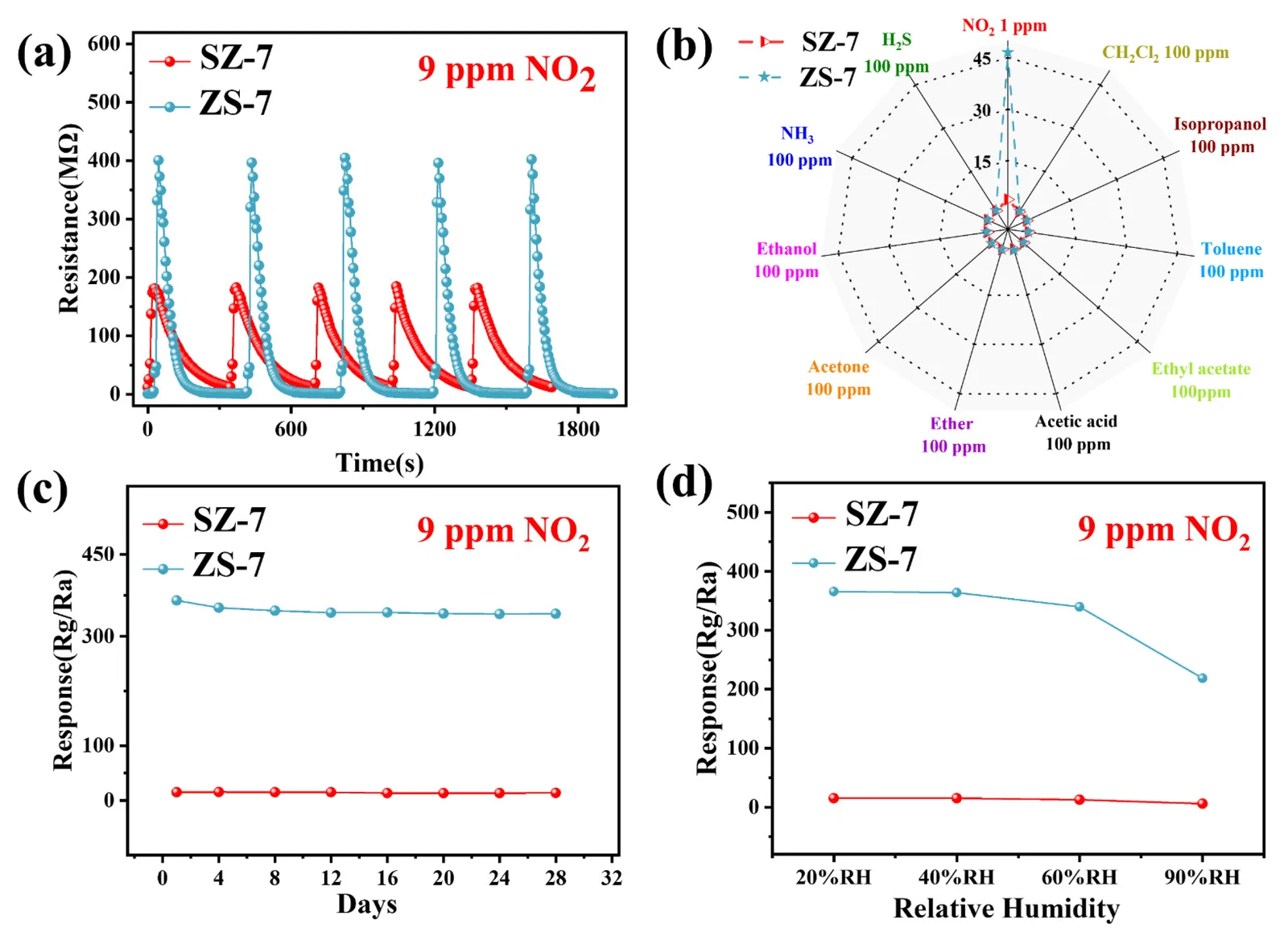
(**a**) Repeatability, (**b**) selectivity, (**c**) long-term stability, and (**d**) response characteristics under different relative humidity conditions of SZ-7 and ZS-7 sensors.

**Figure 11:**
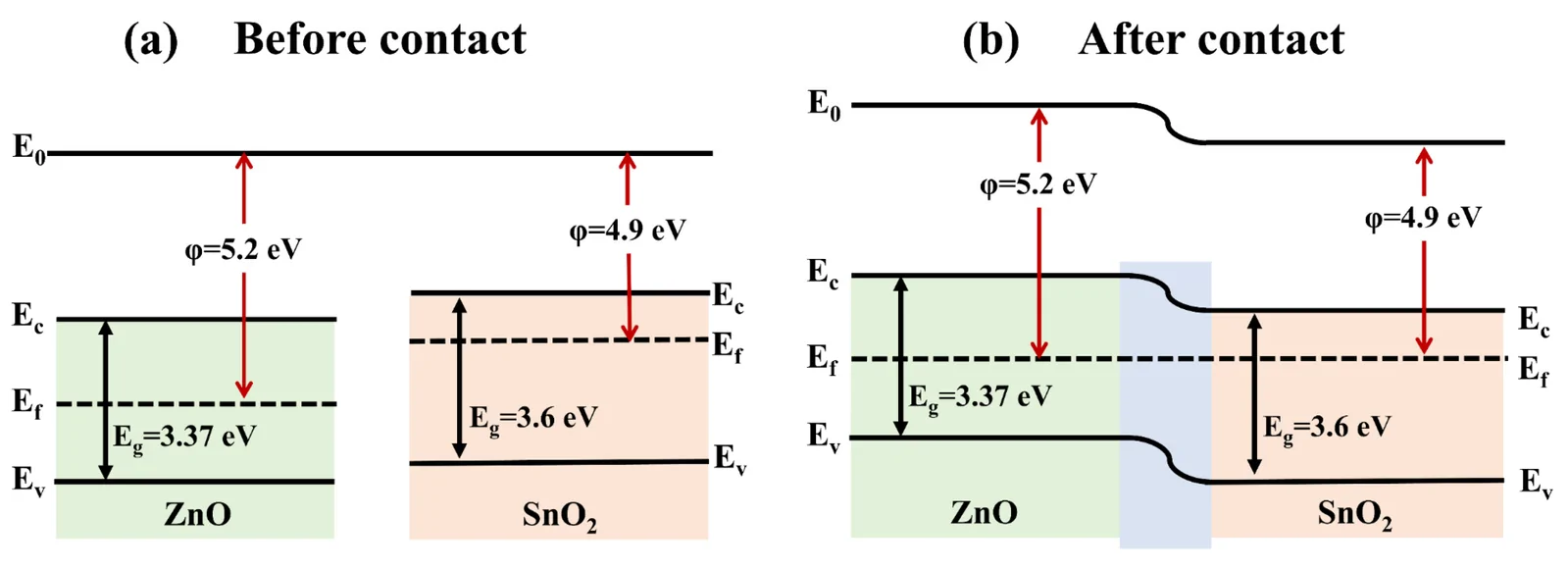
(**a**) Schematic illustration of the energy band structures of pure ZnO and SnO_2_. (**b**) Schematic illustration of the energy band structure of the ZnO/SnO_2_ n–n heterojunction.

**Figure 12:**
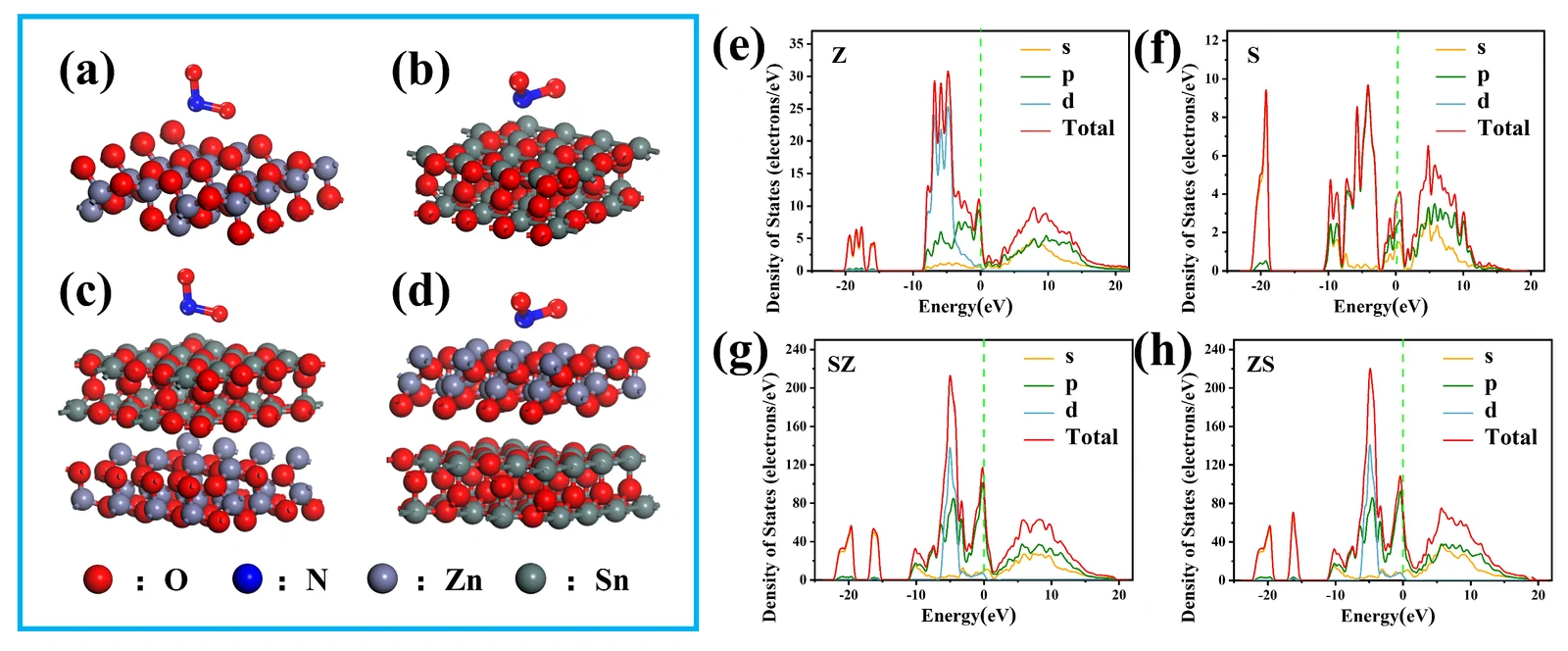
(**a**–**d**) Optimized adsorption models of NO_2_ on the surfaces of Z, S, SZ, and ZS; (**e**) PDOS of Z; (**f**) PDOS of S; (**g**) PDOS of SZ; and (**h**) PDOS of ZS.

**Table 1:** Relative contents of O_C_, O_V,_ and O_L_ in Z, S, SZ-7, and ZS-7.

Sample	O_C_(%)	O_V_(%)	O_L_(%)
Z	11.57	20.26	68.17
S	7.64	11.23	81.13
SZ-7	7.19	26.28	66.53
ZS-7	3.47	16.50	80.03

**Table 2:** Sensing performance comparison of different chemiresistive NO_2_ gas sensors.

Gas-Sensing Material	NO_2_ Concentration (ppm)	Response (R_g_/R_a_)	Response/Recovery Time (s)	Operating Condition	Ref.
SnO_2_/ZnO	0.5	3.36	126/240	RT	[30]
SnO_2_/ZnO	0.1	128	-/-	RT	[31]
SnO/SnO_2_	50	38	32/58	27 °C	[32]
SnO_2_/ZnO	100	7.37	32/-	225 °C	[33]
SZ-7	1	3.68	21/164	254 nm UV-RT	This work
ZS-7	1	46.49	35/122	254 nm UV-RT	This work

## Data Availability

The required data will be provided upon request.

## Supplementary Materials

The following supporting information can be downloaded at: https://www.mdpi.com/article/10.3390/mi17101115/s1. Figure S1: (a) Resistance variation curves of the SZ-7 sensor under UV light on/off conditions. (b) Resistance variation curves of the ZS-7 sensor under UV light on/off conditions. Figure S2: (a) Baseline resistance of the sensors under 254 nm UV irradiation; (b–i) dynamic resistance response curves of the sensors toward different concentrations of NO_2_;. Table S1: Summary of BET parameters for samples Z, S, SZ-7, and ZS-7. Table S2: Linear fitting correlation coefficients (R^2^) and sensitivities (S) of Z, S, SZ-3, SZ-7, SZ-11, ZS-3, ZS-7, and ZS-11. Table S3: Response characteristics of SZ-7 and ZS-7 samples toward various interfering gases.

## References

[1] Huangfu P., Atkinson R. (2020). Long-term exposure to NO_2_ and O_3_ and all-cause and respiratory mortality: A systematic review and meta-analysis. Environ. Int..

[2] Yun H., Ahn S., Oh J., Kang C., Kim A., Kwon D., Ahn S., Park J., Park J., Kim E. (2024). Short-term exposure to outdoor nitrogen dioxide and respiratory mortality, with high-risk populations: A nationwide time-stratified case-crossover study. BMC Public Health.

[3] Amaducci A., Downs J.W. (2023). Nitrogen Dioxide Toxicity.

[4] Hesterberg T.W., Bunn W.B., McClellan R.O., Hamade A.K., Long C.M., Valberg P.A. (2009). Critical review of the human data on short-term nitrogen dioxide (NO_2_) exposures: Evidence for NO_2_ no-effect levels. Crit. Rev. Toxicol..

[5] Lv M.S., Li Y.N., Chen G.L., Gao R., Zhang X.F., Deng Z.P., Xu Y.M., Huo L.H., Gao S. (2024). Mesoporous In_2_O_3_/ZnO heterogeneous microtubes replicated from waste willow catkins for high response and rapid detection of NO_2_ gas at low temperature. Sens. Actuators B Chem..

[6] Liu X., Ma T., Pinna N., Zhang J. (2017). Two-Dimensional Nanostructured Materials for Gas Sensing. Adv. Funct. Mater..

[7] Zhu J., Chen L., Ni W., Cheng W., Yang Z., Xu S., Wang T., Zhang B., Xuan F. (2025). NiO/ZnO Nanocomposites for Multimodal Intelligent MEMS Gas Sensors. ACS Sens..

[8] Liu Z.L., Yang Z., Liu B., Chen Y.N., Feng W. (2024). Construction of Hybrid ZnO/SnO_2_ N–N Heterojunction with Hierarchical Porous Biomorphic Nanostructure as a High-Response Sensor for Methanol Gas. Crystals.

[9] Li B., Zhou Q., Peng S., Liao Y. (2020). Recent advances of SnO_2_-based sensors for detecting volatile organic compounds. Front. Chem..

[10] Wang C., Yin L., Zhang L., Xiang D., Gao R. (2010). Metal oxide gas sensors: Sensitivity and influencing factors. Sensors.

[11] Wang Z., Li P., Feng B., Feng Y., Cheng D., Wei J. (2024). Wireless gas sensor based on the mesoporous ZnO–SnO_2_ heterostructure enables ultrasensitive and rapid detection of 3-methylbutyraldehyde. ACS Sens..

[12] Franco M.A., Conti P.P., Andre R.S., Correa D.S. (2022). A review on chemiresistive ZnO gas sensors. Sens. Actuators Rep..

[13] Shah S., Sajid M., Hassan M., Muhammad Z., Zhao X., Hussain S., Ashurov M., Gohar O., Pan X. (2026). High-performance and humidity-resistant NO_2_ gas sensors using ZnO/SnO_2_ Nano-heterostructures: Experimental and DFT study. Microchem. J..

[14] Lou C., Yang C., Zheng W., Liu X., Zhang J. (2021). Atomic layer deposition of ZnO on SnO_2_ nanospheres for enhanced formaldehyde detection. Sens. Actuators B Chem..

[15] Ren X., Xu Z., Zhang Z., Tang Z. (2022). Enhanced NO_2_ Sensing Performance of ZnO-SnO_2_ Heterojunction Derived from Metal-Organic Frameworks. Nanomaterials.

[16] Er I.K., Uysal S., Ateş A., Acar S. (2025). ZnO/SnO_2_ based composite heterostructure for NO_2_ gas sensing properties. Ceram. Int..

[17] Yang S., Lei G., Xu H., Lan Z., Wang Z., Gu H. (2021). Metal Oxide Based Heterojunctions for Gas Sensors: A Review. Nanomaterials.

[18] Zhang J., Qin Z., Zeng D., Xie C. (2017). Metal-oxide-semiconductor based gas sensors: Screening, preparation, and integration. Phys. Chem. Chem. Phys..

[19] Guo M., Wang B., Bian H., Tao Z., Luo X., Cui Y., Huang J., Tu P. (2025). Low-temperature ppm-level H_2_S flexible gas sensor on the basis of Ag-modified ZnO. Mater. Sci. Semicond. Process..

[20] Chowdhury N.K., Bhowmik B.J.N.A. (2021). Micro/nanostructured gas sensors: The physics behind the nanostructure growth, sensing and selectivity mechanisms. Nanoscale Adv..

[21] Wang W., Fang J., Fan Y., Wang Q., Chen Y., Ruan S. (2024). Regulation of surface oxygen (OV, OC) and enhancement of triethylamine sensing performance of MOF-derived Co_3_O_4_ hollow nanocubes by g-C_3_N_4_ coupling. Sens. Actuators B Chem..

[22] Li G., Zhang H., Meng L., Sun Z., Chen Z., Huang X., Qin Y. (2020). Adjustment of oxygen vacancy states in ZnO and its application in ppb-level NO_2_ gas sensor. Sci. Bull..

[23] Thommes M., Kaneko K., Neimark A.V., Olivier J.P., Rodriguez-Reinoso F., Rouquerol J., Sing K.S. (2015). Physisorption of gases, with special reference to the evaluation of surface area and pore size distribution (IUPAC technical report). Pure Appl. Chem..

[24] Bai X., Wang Y., Gui L., Tao M., Zeng M. (2025). Comparing the Influences on NO_2_ Changes in Terms of Inter-Annual and Seasonal Variations in Different Regions of China: Meteorological and Anthropogenic Contributions. Remote Sens..

[25] Singh A., Mohapatra S., Tomar M., Chowdhuri A., Singh V. (2025). Low temperature NO_2_ gas sensing achieved by mesoporous SnO_2_. J. Alloys Compd..

[26] Chang X., Li S., Chu D. (2020). Sensing of Oxygen Partial Pressure in Air with ZnO Nanoparticles. Sensors.

[27] Lin Z., Li N., Chen Z., Fu P. (2017). The effect of Ni doping concentration on the gas sensing properties of ni doped SnO_2_. Sens. Actuators B Chem..

[28] Huang J., Liang H., Ye J., Jiang D., Sun Y., Li X., Geng Y., Wang J., Qian Z., Du Y. (2021). Ultrasensitive formaldehyde gas sensor based on Au-loaded ZnO nanorod arrays at low temperature. Sens. Actuators B Chem..

[29] Verma G., Kadiri H., Gokarna A., Kumar S., Kumar M., Lerondel G., Gupta A. (2025). Flexible hybrid nanostructured chemiresistive sensing platform for low-temperature trace hydrogen detection. Sens. Actuators B Chem..

[30] Guo J., Li W., Zhao X., Hu H., Wang M., Luo Y., Xie D., Zhang Y., Zhu H. (2021). Highly Sensitive, Selective, Flexible and Scalable Room-Temperature NO_2_ Gas Sensor Based on Hollow SnO_2_/ZnO Nanofibers. Molecules.

[31] Duoc V.T., Hung C.M., Nguyen H., Van Duy N., Van Hieu N., Hoa N.D. (2021). Room temperature highly toxic NO_2_ gas sensors based on rootstock/scion nanowires of SnO_2_/ZnO, ZnO/SnO_2_, SnO_2_/SnO_2_ and, ZnO/ZnO. Sens. Actuators B Chem..

[32] Wang P., Ge W., Jia X., Huang J., Zhang X., Lu J. (2022). SnO/SnO_2_ heterojunction: An alternative candidate for sensing NO_2_ with fast response at room temperature. Front. Mater. Sci..

[33] Kannan S., Pandey G., Lawaniya S.D., Kumar S., Sharma P., Awasthi K. (2024). Metal–Organic Framework (MOF)-Derived SnO_2_–ZnO Nanocomposites for Highly Sensitive NO_2_ Detection. J. Electron. Mater..

[34] Walker J.M., Akbar S.A., Morris P.A. (2019). Synergistic effects in gas sensing semiconducting oxide nano-heterostructures: A review. Sens. Actuators B Chem..

[35] Park J.Y., Choi S.W., Kim S.S. (2011). A model for the enhancement of gas sensing properties in SnO_2_–ZnO core-shell nanofibres. J. Phys. D Appl. Phys..

[36] Wang L., Li J., Wang Y., Yu K., Tang X., Zhang Y., Wang S., Wei C. (2016). Construction of 1D SnO_2_–coated ZnO nanowire heterojunction for their improved n-butylamine sensing performances. Sci. Rep..

[37] Ou L.X., Liu M.Y., Zhu L.Y., Zhang D.W., Lu H.L. (2022). Recent Progress on Flexible Room-Temperature Gas Sensors Based on Metal Oxide Semiconductor. Nano-Micro Lett..

[38] Choi S., Yoon J., Kim T., Bae J.H., Kang Y.G., Park H., Park H.S., Kim J., Lee W.Y., Jang J. (2025). Enhanced mobility and bias stability of SnO_2_ thin-film transistors enabled by Ga_2_O_3_ passivation-induced two-dimensional electron gas. Mater. Today Adv..

[39] Xuan J., Zhao G., Sun M., Jia F., Wang X., Zhou T., Yin G., Liu B. (2020). Low-temperature operating ZnO-based NO_2_ sensors: A review. RSC Adv..

[40] Wang X., Yang X. (2022). General hydrogen gas sensing mechanism on SnO_2_ (110) surface based on DFT simulations. Surf. Sci..

[41] Palai A., Panda N.R., Sahoo M.R., Sahu D. (2022). Study on the electronic band structure of ZnO–SnO_2_ heterostructured nanocomposites with mechanistic investigation on the enhanced photoluminescence and photocatalytic properties. J. Mater. Sci. Mater. Electron..

[42] Zhao K., Xia P., Cao J. (2026). Atomic-scale mechanisms of enhanced NO_2_ adsorption and sensing on ZnO/SnO_2_ heterojunction: A DFT study. Chem. Phys. Lett..

[43] Ganesh Moorthy S., Bouvet M. (2024). Effects of Visible Light on Gas Sensors: From Inorganic Resistors to Molecular Material-Based Heterojunctions. Sensors.

[44] Chizhov A., Rumyantseva M., Gaskov A. (2021). Light Activation of Nanocrystalline Metal Oxides for Gas Sensing: Principles, Achievements, Challenges. Nanomaterials.

