# Supplementary material for: Effect of Different Dominant Phase on NO2-Sensing Properties of ZnO/SnO2 Composites
Source: Micromachines (Basel). 2026 Sep 24;17(10):1115. doi: 10.3390/mi17101115 (PMC13707037; doi:10.3390/mi17101115)
Supplement: Supplementary file 1 [file micromachines-17-01115-s001.zip › micromachines-4537281-supplementary.pdf]

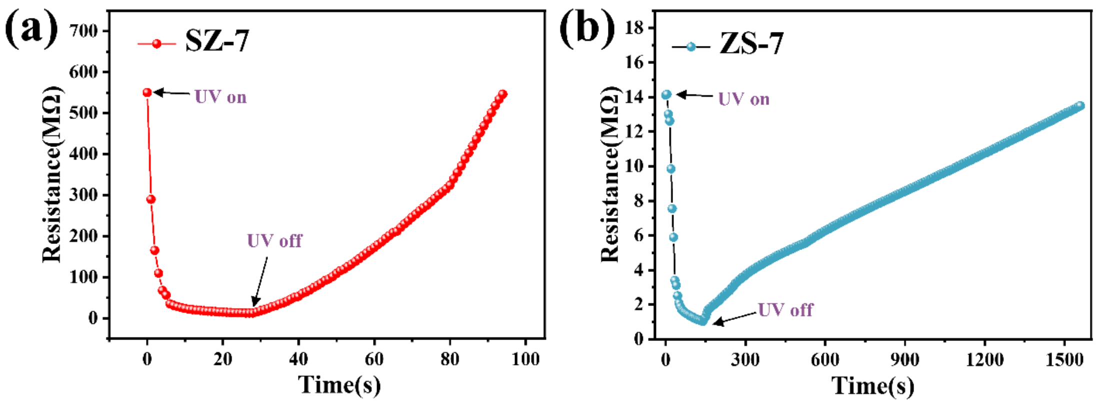

Figure S1. (a) Resistance variation curves of the SZ-7 sensor under UV light on/off conditions. (b) Resistance variation curves of the ZS-7 sensor under UV light on/off conditions.

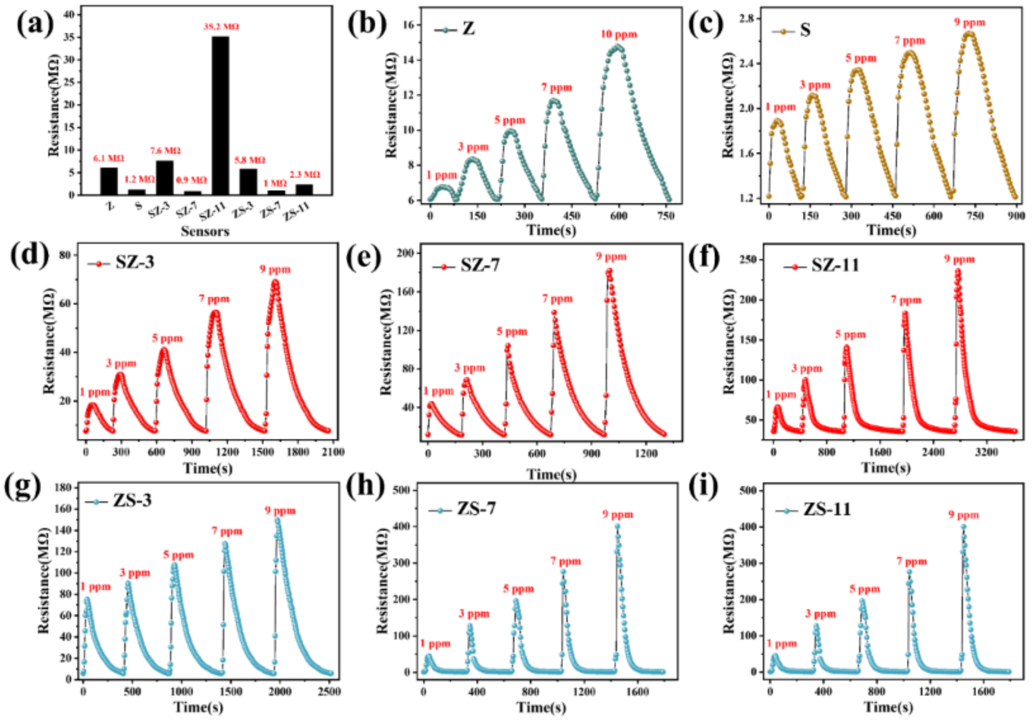

Figure S2. (a) Baseline resistance of the sensors under 254 nm UV irradiation; (b–i) dynamic resistance response curves of the sensors toward different concentrations of NO_2.

Table S1. Summary of BET parameters for samples Z, S, SZ-7, and ZS-7.

Sample	Specific Surface Area (m^2/g)	Total Pore Volume (cm^3/g)	Average Pore Diameter (nm)
**Z**	20.1	0.074	12.61
**S**	145.8	0.088	2.94
**SZ-7**	47.5	0.144	10.84
**ZS-7**	70.3	0.122	5.65

Table S2. Linear fitting correlation coefficients (R^2) and sensitivities (S) of Z, S, SZ-3, SZ-7, SZ-11, ZS-3, ZS-7, and ZS-11.

Samples	R^2	S
**Z**	0.996	0.145
**S**	0.991	0.080
**SZ-3**	0.995	0.818
**SZ-7**	0.992	1.434
**SZ-11**	0.995	0.590
**ZS-3**	0.989	1.613
**ZS-7**	0.998	39.446
**ZS-11**	0.992	2.998

Table S3. Response characteristics of SZ-7 and ZS-7 samples toward various interfering gases.

Interfering gas(es)	SZ-7(R_a/R_g or R_g/R_a)	ZS-7(R_a/R_g or R_g/R_a)
**1 ppm Nitrogen dioxide**	**3.68**	**46.49**
100 ppm Hydrogen sulfide	1.3952	1.4285
100 ppm Ammonia	1.4362	1.5341
100 ppm Ethanol	1.0423	1.2541
100 ppm Acetone	1.1285	1.3025
100 ppm Ether	1.0898	1.1586
100 ppm Acetic acid	1.0342	1.3452
100 ppm Ethyl acetate	1.0134	1.0563
100 ppm Toluene	1.1275	1.0324
100 ppm Isopropanol	1.0272	1.0952
100 ppm Dichloromethane	1.0123	1.2534
